# Acute myocardial infarction at a district hospital in KwaZulu-Natal – Management and outcomes

**DOI:** 10.4102/safp.v64i1.5463

**Published:** 2022-06-13

**Authors:** Zakariya Badat, Selvandran Rangiah

**Affiliations:** 1 Department of Family Medicine, College of Health Sciences, University of KwaZulu-Natal, Durban, South Africa

**Keywords:** acute myocardial infarction, ischaemic heart disease, management outcomes, district hospital, STEMI, NSTEMI

## Abstract

**Background:**

Acute myocardial infarction (AMI) following ischaemic heart disease (IHD) is associated with increased morbidity and mortality. The condition remains a management challenge in resource-constrained environments. This study analysed the management and outcomes of patients presenting with AMI at a district hospital in KwaZulu-Natal.

**Methods:**

A descriptive study that assessed hospital records of all patients diagnosed with AMI over a 2-year period (01 August 2016 to 31 July 2018). Data extracted recorded patient demographics, risk factors, timing of care, therapeutic interventions, follow up with cardiology and mortality of patients.

**Results:**

Of the 140 patients who were admitted with AMI, 96 hospital records were analysed. The mean (standard deviation [s.d.]) age of patients was 55.8 (±12.7) years. Smoking (73.5%) and hypertension (63.3%) were the most prevalent risk factors for patients with ST elevation myocardial infarction (STEMI) in contrast to dyslipidaemia (70.2%) and hypertension (68.1%) in patients with non-ST elevation myocardial infarction (NSTEMI). Almost 49.5% of patients arrived at hospital more than 6 h after symptom onset. Three (12.5%) patients received thrombolytic therapy within the recommended 30-min time frame. The mean triage-to-needle time was 183 min – range (3; 550). Median time to cardiology appointment was 93 days. The in-hospital mortality of 12 deaths considering 140 admissions was 8.6%.

**Conclusion:**

In a resource-constrained environment with multiple systemic challenges, in-hospital mortality is comparable to that in private sector conditions in South Africa. This entrenches the role of the family physician. There is need for more coordinated systems of care for AMI between district hospitals and tertiary referral centres.

## Background

Ischaemic heart disease (IHD) remains a major public health issue and was the leading cause of death globally for every consecutive year between 2000 and 2019.^1^ In 2019, close to 8.9 million deaths were linked to IHD globally.^1^ South Africa has, in the recent years, experienced an increase in mortality from non-communicable diseases (NCD) with 59.3% of deaths falling in this category.^2,3^ Ischaemic Heart Disease is a major contributor to NCD and has consistently appeared as one of the top 10 causes of death in the country.^2^

Acute myocardial infarction (AMI) is defined pathologically as myocardial necrosis, a consequence of prolonged myocardial ischaemia.^4^ Sensitive and specific cardiac enzymes such as cardiac troponins, components of the contractile apparatus of the myocyte, and the MB fraction of creatine kinase (CKMB) are increased.^4^ Clinically, patients with AMI can be subdivided into ST elevation myocardial infarction (STEMI) and non-ST elevation myocardial infarction (NSTEMI).^5^

The European Society of Cardiology (ESC) has published guidelines for managing patients with AMI.^6,7^ In patients presenting with STEMI and symptom onset ≤ 12 h, timely reperfusion, preferably with primary percutaneous coronary intervention (pPCI), is recommended. The door-to-balloon target is < 90 min. In non-PCI capable facilities where delay in performing pPCI of > 120 min is expected, fibrinolytic therapy should be initiated with a door-to-needle time < 30 min as a target. Patients should subsequently be transferred for angiography and revascularisation within 3–24 h.

Immediate transfer should be initiated for patients in cardiogenic shock or heart failure, with urgent transfer for those who have failed reperfusion.^6^ Immediate management of patients with NSTEMI involves risk stratification of patients and tailoring of therapy, both pharmacological and invasive, according to risk category and risk of bleeding. The timing of angiography is ideally within 24 h of admission.^7^

South Africa is a country with a paucity of healthcare resources and unequal distribution of health services in the public and the private sectors.^8^ Seventy five percent of catheter labs are in the private healthcare sector and service only 25% of the population.^8^ The ‘stent for life’ initiative supported by the ESC is looking at ways that patients can have early and easy access to primary pPCI.

In the KwaZulu-Natal public sector, patient care is a huge challenge as only two facilities in the province are PCI equipped. Inkosi Albert Luthuli Central Hospital based in Durban and Grey’s Hospital based in Pietermaritzburg serves the entire region as well as parts of the Eastern Cape with a total population exceeding 11.6 million.^9^

The South African district-based health system provides a network of facilities that connects different levels of care to streamline services. Local hospitals, where family physicians play a major role, manage a wide diversity of patients, including those presenting with acute coronary syndromes (ACS). The outcome on morbidity and mortality is unknown due to a paucity of epidemiologic data on AMI.^10^ Currently, the pharmaco-invasive strategy is the treatment of choice for patients with STEMI, when indicated in the public health sector of KwaZulu-Natal, owing to the lack of resources.^5^

This study analysed the management of patients presenting with STEMI and NSTEMI as well as the outcomes in a district-level resource-limited environment with no PCI or on-site cardiology service.

## Methods

### Study design

This was a descriptive cross-sectional study that assessed hospital records of all patients diagnosed with AMI over a 2-year period from 01 August 2016 to 31 July 2018.

### Setting

The study was conducted at Wentworth Hospital (WWH) in Durban, KwaZulu-Natal. The hospital is a district-level facility that has 230 beds and serves the Durban South catchment area with an estimated population of 407 000 as at 2013.^11^

### Study population and sampling strategy

The hospital records of all patients either admitted with AMI or managed in the Accident and Emergency unit for the condition were assessed. The ESC criteria for diagnosis of AMI as well as age > 18 years were parameters used to include participants.^6,7^

The hospital records checked were the admissions, the discharge as well as the death notification registers in both the High Care and Accident and Emergency units. During the study period, 140 patients were admitted with a diagnosis of AMI. A total of 96 outpatient medical records were traced. Of these, 44 records were either not found or had incomplete notes. This may be due to poor record-keeping and file administration processes at the institution. All deaths were traced through a death notification register. In-patient notes were available to confirm the diagnosis of AMI and assess immediate outcome at discharge. These records had insufficient data to describe outpatient care and hence was excluded from the process of care analyses but included to calculate the in-hospital mortality rate.

Of the 96 patients, seven were admitted twice for recurrent myocardial infarction. Total admissions were thus 103. For process of care, each admission was regarded as a separate instance, as an individual process has an impact on the outcome. However, for patient demographics, mortality rate and days to referral to central hospital with cardiology service, the patient numbers were used in place of admissions to avoid duplicating data.

### Data analysis

All data were collected using a data collection tool that assessed patient demographics, risk factors for IHD, symptoms on presentation, time to Electrocardiogram (ECG) and time to fibrinolysis if indicated. The use of relevant life-saving drugs during high care admission, time taken to follow up with cardiology, and in-hospital mortality of patients with AMI were also tracked. Information was stored electronically on a hard drive in an encrypted file and password protected.

The following statistical parameters were used to determine an appropriate sample size with 80% statistical power. Type 1 error = 0.05 (the probability of rejecting the null hypothesis) which is regardless of the profile of patients there should be no difference in survival. Type 2 error (β) = 0.2 (probability of falsely accepting the null hypothesis). The assumption was that the sample affects the normal distribution, that is, population mean = 0 and σ = 1. A critical *z*-value = 1.95 was used.

Based on the stated statistical parameters, a sample size of 73 was determined to be sufficient to provide 80% statistical power. Continuous variables were summarised and mean ± standard deviation (s.d.) and medians and interquartile ranges were used for highly skewed variables with prominent asymmetrical outliers. Categorical variables were summarised into proportions and percentages and compared using Pearson’s chi square and Fischer’s exact test as appropriate. IBM Statistical Package for Social Sciences version 25 (SPSS Inc., Chicago, IL, United States [US]) was used to analyse the data. A *p*-value of < 0.05 was considered as statistically significant. Associations between categorical predictors and outcomes were assessed using Pearson’s chi square tests or Fisher’s exact tests. Independent samples *t*-tests applied when the predictor was continuous and normally distributed. Multiple logistic regression analysis served to assess odds ratios of exposures for in-hospital mortality while controlling confounding variables. Odds ratios and 95% confidence intervals were reported.

## Results

### Demographics and risk factor profile

Patient characteristics and risk factor profile are presented in Table 1. The mean age of patients was 58 years (Range: 29–87). The mean age for women was 61 (Range: 35–85) and for men 57 years (Range: 29–87). There was slightly higher likelihood of men having STEMI but this was not significant (*p* = 0.2). A borderline of non-significantly younger people had STEMI (*p* = 0.07). White people and mixed-race people were more likely to have STEMI than black people and Indian people but the difference was not statistically significant. Indian people were the least likely to have STEMI of all the race groups. There were no statistically significant variations in risk factors when comparing STEMI and NSTEMI. Smoking (73.5%) and hypertension (63.3%) were the most frequently observed risk factors for patients with STEMI, while dyslipidaemia (70.2%) and hypertension (68.1%) were most common in patients with NSTEMI.

**TABLE 1:** Demographics and risk factor profile of patients with acute myocardial infarction.

Characteristics	STEMI (*n* = 49)			NSTEMI (*n* = 47)			All patients (*n* = 96)			Odds of STEMI compared to NSTEMI		
	*n*	%	Mean ± s.d.	*n*	%	Mean ± s.d.	*n*	%	Mean ± s.d.	OR	95% CI	*p*
**Demographics**												
Female	12	24.5	-	17	36.2	-	29	30.2	-	0.60	0.2–1.4	0.20
Male	37	75.5	-	30	63.8	-	67	69.8	-	1.75	0.7–4.2	0.20
Age	-	-	55.8 ± 12.7	-	-	60.6 ± 12.6	-	-	58.2 ± 12.8	1.03	1.0–1.1	0.07
≥ 50	32	65.3	-	40	85.1	-	72	75.0	-	0.33	0.1–0.9	0.03
**Race**												
Black people	5	10.2	-	4	8.5	-	9	9.4	-	1.20	0.3–4.9	0.80
Indian people	23	46.9	-	32	68.1	-	55	57.3	-	0.40	0.2–0.9	0.04
White people	15	30.6	-	8	17.0	-	23	24.0	-	2.10	0.8–5.7	0.10
Mixed race people	6	12.2	-	3	6.4	-	9	9.4	-	2.00	0.5–8.7	0.30
**Risk factors**												
Previous IHD	11	22.4	-	13	27.7	-	24	25.0	-	0.75	0.3–1.9	0.60
Family history of IHD	19	38.8	-	14	29.8	-	3	32.0	-	1.50	0.6–3.5	0.40
Dyslipidaemia	28	57.1	-	33	70.2	-	61	63.5	-	0.50	0.2–1.3	0.20
Hypertension	31	63.3	-	32	68.1	-	63	65.6	-	0.80	0.3–1.9	0.60
Diabetes mellitus	22	44.9	-	24	51.1	-	46	47.9	-	0.80	0.3–1.7	0.50
Smoking	36	73.5	-	26	55.3	-	62	64.6	-	2.20	0.9–5.3	0.07

OR, odds ratio; CI, confidence interval; STEMI; ST Elevation Myocardial Infarction; NSTEMI, Non-ST Elevation Myocardial Infarction.

### First medical contact parameters

The timing, clinical parameters and immediate management of patients with AMI are presented in Table 2. For 18 (19.0%) of the patients, no arrival mode documentation was available. Most patients with AMI (56.0%) used their own transport. A higher proportion of patients with NSTEMI were transported independently compared to patients with STEMI [73.1% vs 45.1%, odds ratio [OR]: 0.3, 95% confidence interval [CI]: 0.1 – 0.7); *p* = 0.005]. Just over 23.0% of patients first went to a private general practitioner (GP) prior to arriving at hospital. At least 49.5% of attended patients arrived at the care facility more than 6 h after the onset of symptoms; there was no statistically significant associations between the type of AMI. Electrocardiogram time within 10 min of arrival was achieved in a total of 17 (16.5%) admissions. Majority of the ECG’s – 42 (40.8%) after arrival and triage, took more than 30 min to acquire. Almost 10.7% of patients were sent home, inappropriately discharged on first medical contact (FMC) by the attending general practitioners.

**TABLE 2:** Timing, clinical parameters, and immediate management of patients with acute myocardial infarction.

Characteristics	STEMI (*n* = 51)			NSTEMI (*n* = 52)			All admissions (*n* = 103)			Odds of STEMI compared to NSTEMI		
	*n*	%	Mean ± s.d.	*n*	%	Mean ± s.d.	*n*	%	Mean ± s.d.	OR	95% CI	*p*
**Mode of arrival**												
Own transport	23	45.1	-	38	73.1	-	61	56.0	-	0.3	0.1–0.7	0.005
Private ambulance	3	5.9	-	4	7.7	-	7	7.0	-	0.8	0.2–3.5	0.716
Public ambulance	11	21.6	-	6	11.5	-	17	18.0	-	2.1	0.7–6.2	0.176
Unknown	14	27.5	-	4	7.7	-	18	19.0	-	4.5	1.3–14.9	0.013
**First medical contact**												
Public hospital	38	74.5	-	35	67.3	-	73	70.9	-	1.4	0.6–3.3	0.422
Clinic	1	2.0	-	1	1.9	-	2	1.9	-	1.0	0.1–16.8	0.989
Private GP	10	19.6	-	14	26.9	-	24	23.3	-	0.7	0.3–1.7	0.382
Private hospital	1	2.0	-	2	3.8	-	3	2.9	-	0.5	0.0–5.7	0.577
Unknown	1	2.0	-	0	0.0	-	1	1.0	-	3.1	0.1–78.4	-
**Time from symptom onset to district hospital**												
Unknown	4	7.8	-	3	5.8	-	7	6.8	-	1.4	0.3–6.5	0.677
0–6 h	25	49.0	-	20	38.5	-	45	43.7	-	1.5	0.7–3.4	0.281
> 6–12 h	3	5.9	-	5	9.6	-	8	7.8	-	0.6	0.1–2.6	0.483
> 12–24 h	12	23.5	-	6	11.5	-	18	17.5	-	2.4	0.8–6.9	0.115
> 24 h	7	13.7	-	25	24.3	-	25	24.3	-	0.3	0.1–0.7	0.007
**Time from triage to ECG acquisition**												
≤ 10 min	6	11.8	-	11	21.2	-	17	16.5	-	0.5	0.2–1.5	0.205
> 10–30 min	10	19.6	-	4	7.7	-	14	13.6	-	2.9	0.9–10.0	0.088
> 30 min	21	41.2	-	21	40.4	-	42	40.8	-	1.0	0.5–2.3	0.935
Unknown	6	11.8	-	6	11.5	-	12	11.7	-	1.0	0.3–3.4	0.972
Prehospital ECG	8	15.7	-	10	19.2	-	18	17.5	-	0.8	0.3–2.2	0.636
**Clinical parameters**												
Prehospital troponin available	1	2.0	-	6	11.5	-	7	6.8	-	0.2	0.02–1.3	0.088
Sent home on FMC	5	9.8	-	6	11.5	-	11	10.7	-	0.8	0.2–2.9	0.776
SBP	-	-	136 ± 32.6	-	-	135 ± 29	-	-	136 ± 30.7	-	-	0.89
DBP	-	-	88.7 ± 22	-	-	84.9 ± 19.9	-	-	86.6 ± 20.9	-	-	0.4
Pulse	-	-	82.5 ± 21.7	-	-	92.9 ± 23.5	-	-	87.7 ± 23.1	-	-	0.02
Atypical chest pain	16	31.4	-	20	38.5	-	36	35.0	-	-	-	0.25
Initial troponin *T*	164	50–618	-	317	75–760	-	180	50–715	-	-	-	< 0.001
Peak troponin *T*	1797	895–2000	-	556	319–1390	-	1069	386–2000	-	-	-	0.02

OR, odds ratio; CI, confidence interval; STEMI; ST Elevation Myocardial Infarction; NSTEMI, Non ST Elevation Myocardial Infarction; SBP, systolic blood pressure; DBP, diastolic blood pressure; ECG, electrocardiogram; FMC, first medical contact.

### Therapeutic interventions

**FIGURE 1:**
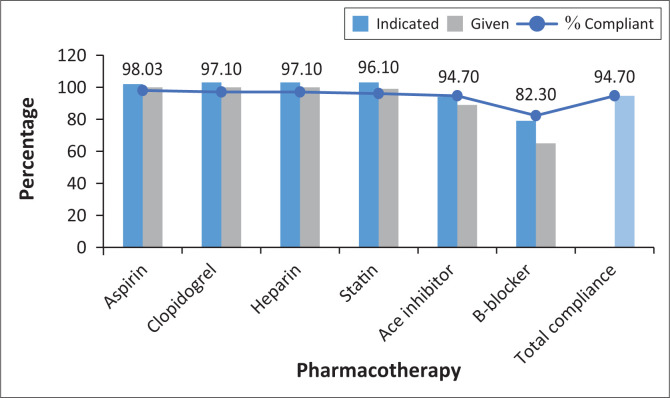
Medical management of acute myocardial infarction.

The therapeutic interventions are demonstrated in Figure 1. Thrombolytic therapy was indicated in 32% (*n* = 33) of admissions. Twenty-four patients (72.7%) received therapy, three of whom (12.5%) were thrombolysed within the 30-min recommended time frame. The mean triage to needle time was 183 min (range: 3–550). There was 94.7% compliance to medical management of AMI with the use of aspirin, clopidogrel, heparin and statins. An angiotensin converting enzyme inhibitor was used in at least 9.05% of patients where indicated (Figure 1). Beta-blocker use had the lowest adherence at 82.3% (Figure 1).

### Outcomes

The clinical outcomes of the study patients are presented in Table 3.

**TABLE 3:** Tertiary involvement and outcomes of patients with acute myocardial infarction.

Characteristics	STEMI (*n* = 49)			NSTEMI (*n* = 47)			All patients (*n* = 96)			Odds of STEMI compared to NSTEMI		
	*n*	%	Mean ± s.d.	*n*	%	Mean ± s.d.	*n*	%	Mean ± s.d.	OR	95% CI	*p*
**Tertiary care**												
Transferred during admission	12	24.5	-	2	4.3	-	14	14.6	-	7.3	1.5–34.7	0.012
Patients booked for cardiology	35	71.4	-	33	70.2	-	68	70.8	-	1.1	0.4–2.6	0.896
Angiography done	16	32.7	-	6	12.8	-	22	22.9	-	3.3	1.2–9.4	0.025
MIBI	22	44.9	-	12	25.5	-	34	35.4	-	2.4	1.0–5.6	0.05
CABG done	5	10.2	-	1	2.1	-	6	6.25	-	-	-	-
**Immediate complications**												
Arrhythmia	6	12.24	-	5	10.6	-	11	11.4	-	1.2	0.3–4.1	0.805
Cardiogenic shock	5	10.20	-	1	2.1	-	6	6.2	-	5.2	0.6–46.5	0.138
Post infarct angina	1	2.0	-	2	4.2	-	3	3.1	-	0.5	0.0–5.3	0.541
Pulmonary oedema	0	0.0	-	2	4.2	-	2	2.1	-	0.2	0.0–3.9	0.279
Recurrent MI	4	8.2	-	3	6.4	-	7	7.3	-	1.3	0.3–6.2	0.738
CCF	4	8.2	-	6	12.8	-	10	10.4	-	0.6	0.2–2.3	0.464
CVA	0	0.0	-	1	2.1	-	1	1.0	-	0.3	0.0–7.9	0.481
LV thrombus	2	4.1	-	0	0.0	-	2	2.1	-	5.0	0.2–107	0.303
Total	22	44.5	-	20	42.5	-	4	43.8	-	1.1	0.5–2.5	0.817
**Immediate in-hospital outcome (including at transfer hospital)**												
Alive	40	81.6	-	44	93.6	-	84	87.5	-	0.3	0.1–1.2	0.089
Demised	9	18.4	-	3	6.4	-	12	12.5	-	3.3	0.8–13.1	0.089
**30-day outcome in patients with follow up investigations†**												
Echo EF	29	-	48.6 ± 7.1	26	-	50.9 ± 11.4	55	-	49.7 ± 9.4	-	-	0.360
EF < 45%	8	27.6	-	6	23.1	-	14	25.5	-	1.2	0.4–3.9	0.768
EF ≥ 45%	21	72.4	-	20	76.9	-	41	74.5	-	0.9	0.4–2.1	0.883
**30-day outcome in patients with follow up – NYHA class** ‡												
Class I	17	44.7	-	14	33.3	-	31	38.8	-	1.3	0.6–3.1	0.488
Class II	14	36.8	-	17	40.5	-	31	38.8	-	0.9	0.4–2.1	0.825
Class III	4	10.5	-	3	7.1	-	7	8.8	-	1.5	0.3–7.0	0.626
Class IV	1	2.6	-	0	0.0	-	1	1.3	-	3.3	0.1–83.7	0.467
Unknown	2	5.3	-	8	19	-	10	12.5	-	0.3	0.1–1.4	0.118
**30-day outcome in patients with follow up – CCS class§**												
Class I	26	68.4	-	23	54.8	-	31	38.8	-	1.3	0.6–2.5	0.540
Class II	8	21.1	-	11	26.2	-	31	38.8	-	0.8	0.3–2.2	0.672
Class III	2	5.3	-	0	0.0	-	7	8.8	-	5.5	0.3–119	0.275
Unknown	2	5.3	-	8	19	-	10	12.5	-	0.3	0.1–1.4	0.118

OR, odds ratio; CI, confidence interval; STEMI; ST Elevation Myocardial Infarction; NSTEMI, Non ST Elevation Myocardial Infarction; MIBI, myocardial perfusion imaging scan; CABG, coronary artery bypass graft; MI, myocardial infarction; CCF, congestive cardiac failure; CVA, cerebrovascular accident; LV, left ventricular; EF, ejection fraction

† , 30-day outcome in patients with follow up investigations: STEMI – *n* = 29, NSTEMI – *n* = 26, All patients– *n* = 55;

‡ , 30-day outcome in patients with follow up – NYHA Class: STEMI – *n* = 38, NSTEMI – *n* = 42, All patients – *n* = 80;

§ , 30-day outcome in patients with follow up – CCS Class: STEMI – *n* = 38, NSTEMI – *n* = 42, All patients – *n* = 80.

Transfer for higher level of care was affected for 24.5% of patients with STEMI compared to 4.3% with NSTEMI. [OR: 7.3, 95% CI: 1.5 – 34.7, *p* = 0.012].

Fewer than 30% of patients were not booked for cardiology due to frailty, renal dysfunction, or patient refusal. Median time to cardiology appointment was 93 days (range: 0–180 days). Sixty-eight patients (70.8%) were given a cardiology appointment. Time to angiography from index presentation was in the range of (1–193) days with 22.9% undergoing the procedure. A myocardial perfusion imaging scan (MIBI) was done on 35.4% of the patients.

Of those patients who were alive, 55 (55/84, 65.5%) had an echo report. The mean Ejection Fraction (EF) was 49.7% s.d. (45–55.5). Of the 12 inpatient deaths, 18.4% were due to STEMI and 6.4% due to NSTEMI. The mean age was 66 years with a range of 41–87. There was no association between > 12-h delay in presentation and mortality (*p* = 0.918). There was statistically significant association between SBP < 100 and mortality (*p* < 0.001). Those with SBP < 100 were more likely to die. Increasing age was significantly associated with in-hospital mortality (*p* = 0.002) with those who died being older. There was an association between peak troponin *T* being > 2000 and mortality (*p* < 0.001).

The odds of death in hospital were 3.3 times higher in STEMI than in NSTEMI (*p* = 0.089). After controlling for confounding effects of age and SBP, the odds of death in hospital emerged 6.2 times higher in STEMI than NSTEMI (*p* = 0.039). A multivariable analysis for factors associated with death or major cardiovascular complications by logistic regression was done. After multivariable adjustment, diagnosis with STEMI, (OR: 4.23; 95% CI: 1.31–13.67; *p* = 0.02), delay in presentation of 12–24 h from symptom onset (OR: 0.16; 95% CI: 0.04–0.70; *p* = 0.02), smoking (OR: 0.26; 95% CI: 0.08–0.90; *p* = 0.03), previous IHD (OR: 6.82; 95% CI: 2.06–22.61; *p* = 0.002) and initial troponin *T* > 2000 (OR: 7.18; 95% CI: 2.23–23.12; *p* = 0.04) were significantly associated with death or major cardiovascular complications.

## Discussion

The demographics describing this cohort of patients are similar to other centres in KwaZulu-Natal, where Indian people factor as the major race group at risk for AMI.^12,13,14,15,16^ A study in KwaZulu-Natal, demonstrated an incidence of 56% of patients with STEMI and 63% of patients with NSTEMI in the Indian population at Northdale hospital in Pietermaritzburg.^12^ Other data indicated that 94.4% of patients were Indian while only 5.6% were black people.^13^ The authors commented on the influence of the surrounding population on the number of Indian patients in the study. The white and mixed race populations are represented at WWH in comparison to other centres in the eThekwini and uMgungundlovu districts.

The lower incidence of AMI in women is consistent with international findings. In the United States (US) men were 6.8 years younger than women with an average age of 65.0 years at first AMI for men compared to 71.8 years for women.^17^ Developing, middle eastern and Asian countries, Qatar, Lebanon and Iran report AMI a decade earlier than the US.^18^ India, Pakistan, Sri Lanka, Nepal and Bangladesh show a lower average age of first MI – 53.0 years in men and 58.6 years in women.^19^ The mean age of diagnosis of AMI reported in this study is similar to that in Asian countries at 57 years for men versus 61 years for women.

The data indicates that there is a chasm in developing countries with regard to preventative healthcare. High prevalence of obesity, cigarette smoking, recreational drug use and poor control of comorbidities points to upstream factors that need attention to improve cardiovascular health.^20^ A demographic health survey in 2016 noted that 68% of South African women and 31% of men were obese. Over a third (36%) of men smoke cigarettes, as did 7% of women aged 15 years and over. One in three adults was not interested in lowering salt intake, maintained high intake of sugar and consumed little fruit – 51% did not consume any fruit the day or night before the survey.^20^

The diabetes and hypertension prevalence were 13% and 45%, respectively.^20^ Nearly 90% had uncontrolled hypertension.^20^ A study in *The Lancet Global Health*,^21^ found that southern sub-Saharan Africa has the highest rates of diabetes, cardiovascular disease and substance use disorders within sub-Saharan Africa. These comorbidities are mirrored in the study population for this research, signaling the importance of a holistic approach to cardiovascular health.

A significant proportion of admissions (25%) used emergency medical services to arrive at hospital. Although 23.3% of admissions with AMI were first seen by a private GP, only 17.5% of patients admitted arrived with a prehospital ECG. Patients with NSTEMI (73.1%) were more likely to use own transport to reach the hospital than patients with STEMI (45.1%) (OR: 0.3; 95% CI: 0.1–0.7; *p* = 0.005). This may be due to the delay in presentation – 24.3% of NSTEMI admissions versus 13.7% STEMI admissions presented > 24 h after an event.

Even though not statistically significant, delayed presentation and intervals post FMC impact morbidity and mortality.^22^ Systems of care incorporating all stakeholders, namely, community mobilisation, EMS response, the private GP and the different tiers of hospitals must work in tandem for patients with AMI to achieve timely care.

Comparing door-to-needle times in South Africa, three previous studies showed poor compliance with guidelines. Data was captured for hospitals in three cities. At Steve Biko Hospital in Pretoria, only 3% of patients received thrombolytic therapy within 1 h.^23^ In Cape Town, 20.5% of patients were treated within 30 min,^24^ while at Northdale Hospital in Pietermaritzburg, the mean door-to-needle time was 43 min.^12^ In comparison, in this study, only 12.5% of patients achieved a time within 30 min. Thrombolytic therapy was indicated in 27.3% of patients where it was not given.

A quality improvement project (QIP) addressed this at a rural district hospital by developing a protocol and displaying it in key clinical areas. In-service training with medical and nursing teams focused on recognition, investigation and management of STEMI and off-site ECG interpretation in a setting of clinical uncertainty.^25^ Door-to-needle times improved from 43% baseline to 74% within 6 months post intervention.^25^ Streptokinase causes significant delays and is used for patients at WWH.^26^ Another study concluded that Tenecteplase (TNK) takes 10.5 min less time to prepare than standard treatment (*p* < 0.001) with 18% more patients (*p* < 0.01) meeting target times.^26^ Auditing door-to-needle times and addressing delays can enhance quality assurance.

Medical management is protocol driven; 94.7% of medications indicated were administered. This may account for the unexpected comparable in-hospital mortality to that seen in private sector in South Africa (9.7%).^27^ For the 140 admissions at WWH, in-hospital mortality rate was 8.6%. In contrast, for the US, AMI mortality was lower at 4.6%,^28^ and similar to middle eastern countries like Tunisia – 9.6%.^29^

Resource limitations pose ethical challenges in healthcare service delivery. Only 68% of patients in this study cohort were seen by a cardiologist. After a patient is logged with cardiology, feedback is only given when a consultant has reviewed the case. A minimum of three calls are made before a patient is given an appointment date – usually within three months. The median waiting period of 93 days implies that pharmacologic non-invasive strategy is the status quo at WWH.

Only 22.9% of patients underwent angiography in a 2-year period and only 57.3% had an echo report. The burden of illness calls for the need to realign the health system and mobilise the private sector or alternatively, upskill cardiology fellows to perform interventional cardiology. The odds of death in hospital were 6.2 times higher in STEMI than NSTEMI (*p* = 0.039) after controlling for confounding effects of age and SBP. With limited resources, patients with STEMI should be prioritised for the pharmaco-invasive route with immediate transfer to a cardiology service.

As Gouda and colleagues suggest,^21^ there is urgent need to prepare health services. This study shows health system weaknesses at the coalface and calls for an emergency action plan to deal with current practice. Resource allocation should be according to patient need – having an echo service at a district hospital is necessary if the nature of illness requires it. An obstructive referral pathway leads to higher mortality when patients are made to wait for months for life-saving interventions. This has to be balanced with human resource shortages. Upskilling junior doctors can improve access to healthcare.

This study also highlights the role of family physician in the management of clinical complexity. Family physicians lead a team at the district hospital where, from admission to discharge and follow-up, patients with AMI are managed sometimes without seeing a cardiologist. Although gaps in care are noted, these are correctable with QIPs. The in-hospital mortality of 12 deaths out of 140 admissions was 8.6% compared to the private sector in-hospital mortality of 9.7%. The scientific value of this study is that it reflects the quality of care in patients who have sustained myocardial infarction at a district public hospital. The social value of this study is that it provides a baseline that can influence subsequent changes to policies and guidelines improving overall care of patients with myocardial infarction.

## Limitations

This was a retrospective study and was limited by the data present in patient notes. Of the 140 admissions identified, only 96 could be used for data analysis as 44 records were either missing or had incomplete notes. The large number of missing files stresses the need for upgrade of record-keeping to electronic platforms. This study did not report on long-term outcomes with AMI. There was limited data to calculate BMI, and lack of extensive documentation led to many non-pharmacological interventions not being assessed.

## Conclusion

Despite resource constraints with multiple systemic challenges of a public hospital, where in-hospital care can be provided for AMI patients, mortality is comparable to that in private facilities in South Africa. This entrenches the role of the family physician. There is a need for more coordinated systems of care for AMI between district hospitals and the tertiary referral centres.
